# The short- and long-term outcomes of radical antegrade modular pancreatosplenectomy for adenocarcinoma of the body and tail of the pancreas

**DOI:** 10.1186/s12893-015-0107-0

**Published:** 2015-10-30

**Authors:** Masaaki Murakawa, Toru Aoyama, Masahiro Asari, Yusuke Katayama, Koichiro Yamaoku, Amane Kanazawa, Akio Higuchi, Manabu Shiozawa, Satoshi Kobayashi, Makoto Ueno, Manabu Morimoto, Naoto Yamamoto, Takaki Yoshikawa, Yasushi Rino, Munetaka Masuda, Soichiro Morinaga

**Affiliations:** Department of Gastrointestinal Surgery, Kanagawa Cancer Center, 2-3-2 Nakao, Asahi-Ku, Yokohama, 241-8515 Japan; Department of Gastrointestinal medicine, Kanagawa Cancer Center, Yokohama, Japan; Department of Surgery, Yokohama City University, Yokohama, Japan

**Keywords:** RAMPS, Body and tail pancreatic adenocarcinoma, Pancreatosplenectomy

## Abstract

**Background:**

Radical antegrade modular pancreatosplenectomy (RAMPS) is a relatively new modification of the standard distal pancreatosplenectomy. In this method, dissection proceeds from right-to-left to achieve negative posterior resection margins. However, short-term and long-term outcomes of RAMPS for pancreatic cancer have not yet been clarified. The aim of this study is to evaluate short-term and long-term outcomes in the patients who have undergone RAMPS.

**Methods:**

Consecutive 49 patients were selected from the retrospective database of the Kanagawa Cancer Center from 2000 to 2014. Data from the operative notes, pathology reports, postoperative data, and outpatient data (recurrence and survival) were entered into the database.

**Results:**

All patients were undergone anterior RAMPS. The median operation time was 278 min (range from 140 to 625 mins). The median blood loss in operation was 850 ml (range from 60 to 2790 ml). The overall incidence of morbidity was 51.4 % and the incidence of mortality was 0 %. Forty-one patients (83.7 %) had negative resection margins. The mean number of lymph nodes harvested was 15 and 27 patients had lymph node metastasis. After the median follow-up period was 41.1 months, 1-year and 3-year overall survival rates were 84.1 and 38.6 %, respectively. Median overall survival was 22.6 months.

**Conclusions:**

The present study results suggested that RAMPS procedure might be safe and feasible without an increase in morbidity and morbidity and have survival benefit compared with standard DP.

## Background

Pancreatic cancer, with a five-year survival rate of 5–10 %, is the fourth leading cause of cancer-related death in developed countries [1]. Complete resection is essential for cure. Distal pancreatectomy (DP) has been the standard procedure for the resection of tumors of the body and tail of the pancreas for over 100 years [2].

Adenocarcinoma of the pancreatic body and tail are as aggressive as pancreatic head tumors in terms of local invasion and their propensity for lymph node metastasis. To achieve a cure, it is therefore essential to perform a complete resection of the tumor with a margin of normal tissue and to resect the regional lymph nodes. However, the traditional approach of left-to-right pancreatosplenectomy is associated with a high rate of tangential margin positivity [3, 4], and is not based on the lymphatic drainage of the organ.

Strasberg et al. [2, 3] described an operative technique that allows for a more complete dissection of posterior margin and which incorporates lymph node mapping for the resection of all of the regional nodes. Radical antegrade modular pancreatosplenectomy (RAMPS) is a relatively new modification of the standard distal pancreatosplenectomy procedure. In this method, dissection proceeds from right-to-left in 1 of 2 posterior dissection planes to achieve negative posterior resection margins. The accompanying N1 lymph node dissection is based on the established anatomy of lymph node drainage of this part of the pancreas. Recently, some literatures had been published about surgical outcomes of the RAMPS [5, 6], however, outcomes of RAMPS in the treatment of pancreatic cancer remain to be clarified.

The aim of the present study is to evaluate the short-term and long-term outcomes of 49 patients who underwent RAMPS and to compare the results with the results of previously published surgical case series in which open and laparoscopic procedures were performed in patients with adenocarcinoma of the pancreatic body and tail.

## Methods

### Patients

The patients were selected from the retrospective database of the Kanagawa Cancer Center from 2000 to 2014. The patients were selected according to the following criteria: (1) a histologically proven pancreatic adenocarcinoma located in the body and tail of pancreas according to the seventh edition of the International Union Against Cancer (UICC) TNM [7]; (2) the patients underwent distal pancreatosplenectomy as the primary treatment for pancreatic adenocarcinoma; (3) the patients underwent surgery that consisted of dissection of more than the N1 lymph nodes and which achieved a curative or R1 resection; (4) the patients did not have synchronous or metachronous malignancies. Patients with pancreatic cancer derived from intraductal papillary mucinous neoplasms, mucinous cystic neoplasms, and neuroendocrine tumors were excluded from the present study.

### Procedure

We performed the RAMPS procedure in patients with adenocarcinoma of the pancreatic body and tail. The initial dissection in the RAMPS procedure begins medially. The splenic artery and vein are ligated and the neck of the pancreas is transected. The dissection continues posteriorly to the aorta at the celiac and superior mesenteric trunks. The resection plane is decided based on the progression of cancer. In patients in whom the tumor appears to penetrate the adrenal gland (or more deeply), the plane of dissection is deepened to the posterior plane behind the adrenal gland.

### Perioperative care

In principle, all of the patients received the same perioperative care. In brief, the patients were allowed to eat until midnight on the day before the surgery and were required to drink the contents of two 500-ml plastic bottles containing oral rehydration solution until 3 h before surgery. The nasogastric tube was removed on postoperative day 1 after surgery. Oral intake was initiated on POD 2, beginning with water and an oral nutritional supplement. The patients began to eat solid food on POD 5 (starting with rice gruel and soft food on POD 3 and advancing in three steps to regular food intake on POD 7). The patients were discharged when they had achieved adequate pain relief and soft food intake, had returned to their preoperative level of mobility and exhibited normal laboratory data.

### Adjuvant chemotherapy

Based on the results of CONKO-001 [8], the patients received gemcitabine adjuvant treatment from 2007 to 2011. Treatment with gemcitabine was initiated within eight weeks after surgery. The patients received a weekly dose of 1,000 mg/m^2^ for three weeks, followed by one week of rest. S-1 chemotherapy was started within 10 weeks after surgery. Based on the results of JASPAC-01, the patients received S-1 adjuvant treatment from 2011 to 2014 [9]. The patients received S-1 (40 mg/m^2^ of body-surface area) twice a day for four weeks, followed by two weeks of rest as one course (six-week schedule) or two weeks followed by one week of rest as one course (three-week schedule). In principle, all patients continued gemcitabine or S-1 treatment for six months.

### Follow up

The patients were followed up at outpatient clinics. The levels of the CEA and CA19-9 tumor markers were measured at least every three months for five years. All patients underwent CT examinations every three months during the first three years after surgery; CT examinations were then performed every six months until five years after surgery.

### Evaluations and statistical analysis

Overall survival was calculated from the date of first surgery to the date of death by any cause or the last day of follow-up. The two groups were compared by unpaired Student’s *t*-test or the *Χ*^2^ test. P values of <0.05 were considered to be statistically significant. The data are presented as medians ± ranges. Survival curves were calculated using the Kaplan-Meier method and compared using the log-rank test. This study was approved by the Institutional Review Board of Kanagawa Cancer Center (2015.epidemiologic study 32-1). This study was in compliance with the Declaration of Helsinki.

## Results

### The background of the patients

Forty-nine patients were eligible for inclusion in the present study. The median patient age was 68 years (range: 46-86 years). Thirty-one patients were male, and 16 were female. The median follow-up period was 41.1 months (range: 5.6-98.3 months). Forty-six patients received adjuvant chemotherapy after surgery (gemcitabine adjuvant chemotherapy [*n* = 23], gemcitabine plus S-1 adjuvant chemotherapy [*n* = 1], and S-1 adjuvant chemotherapy [*n* = 22]. Three patients refused to undergo adjuvant chemotherapy.

### Surgical findings and complications

All of the patients underwent anterior RAMPS. The median operation time was 278 min (range: 140–625 min). The median operative blood loss was 850 ml (range: 60–2790 ml). Blood transfusion was required in 11 of 49 patients (22.4 %). Postoperative complications occurred in 20 patients (40.8 %). Table 1 showed the operative findings and surgical complications. The surgical complications were graded according to the Clavien-Dindo classification system [10, 11]: grade 2 complications occurred in 18 patients (36.7 %), a grade 3a complication occurred in one patient (2.1 %), and a grade 3b occurred in one patient (2.1 %). The mean hospital stay was 21 days (range: 8–57 days).

**Table 1:** Operative findings and surgical complications

*Operative findings*		
Operation time (min)		257 (140–625)
Estimated blood loss		610 (60–2790)
Transfusion (%)		6 (15.3 %)
Length of hospital stay (day)		21 (8–57)
*Surgical complications*		
Clavien-Dindo Grade II	Pancreatic fistula	14 (28.6 %)
	Delayed gastric empty	3 (6.1 %)
	Surgical site infection	1 (2.1 %)
Clavien-Dindo Grade IIIa	Intraabdominal abscess	1 (2.1 %)
Clavien-Dindo Grade IIIb	Intraabdominal abscess	1 (2.1 %)
Total		20 (40.8 %)
*Resection of tumor*		
R0		39
R1		10
DPM positive		7/39 (17.9 %)

### Pathological findings

Three patients had T1 tumors, 1 patient had a T2 tumor, 37 patients had T3 tumors and 4 patients had T4 tumors. Twenty-seven of the 49 patients had positive lymph nodes (55 %). According to American Joint Committee on Cancer Staging of Tumors, 3 patients had Stage IA disease, 15 patients had Stage IIA, 23 patients had Stage IIB, and 8 patients had Stage III. The tumors ranged from 5–83 mm in diameter (mean diameter; 38 mm). Thirteen patients had well-differentiated tumors, 22 patients had moderately differentiated tumors, 8 patients had poorly differentiated tumors, 2 patients had anaplastic carcinoma, 2 patients had papillary adenocarcinoma, and 1 patient had mucinous and adenosquamous carcinoma. The mean number of lymph nodes harvested was 15, 27 patients had lymph node metastasis. Forty-one patients (83.7 %) had negative resection margins.

### Overall survival and recurrence free survival

**Fig. 1:**
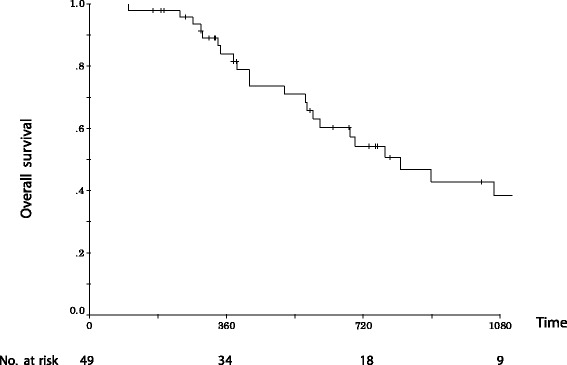
Cumulative survival rates after Radical antegrade modular pancreatosplenectomy (RAMPS) for adenocarcinoma of the pancreatic body and tail

At the time of writing, 25 of the 49 patients (51 %) are alive and 24 patients (49 %) have died. Nineteen of the 25 surviving patients (38.8 %) showed no evidence of recurrence, while 6 (12.2 %) patients are alive with recurrence. The median overall survival was 22.6 months. The 1-year and 3-year overall survival rates were 84.1 and 38.6 %, respectively (Fig. 1). Thirty of 49 patients experienced recurrence. The sites of recurrence included the liver (*n* = 6), the lymph nodes (*n* = 11), the pancreatic bed (*n* = 6), the peritoneum (*n* = 5), and the lungs (*n* = 1). Among 11 lymph nodes recurrences patients, 8 patients recurred in para-aortic lymph node, 3 patients recurred in para-superior mesenteric artery lymph node.

## Discussion

We evaluated the short-term and long-term results of the RAMPS procedure for pancreatic cancer. The overall incidence of morbidity was 51.4 %, while the incidence of mortality was 0 %. Moreover, median survival was 22.6 months and 5-year overall survival was 27 %. These results suggest the safety and feasibility of the RAMPS procedure and indicate the possibility that it could be performed without an increase in morbidity or mortality and that it might offer a survival benefit in comparison to standard DP.

Although postoperative complications occurred in 20 of the 49 patients (40.8 %), there were no postoperative or in-hospital deaths. Pancreatic fistula was the most frequently diagnosed complication, followed by abdominal abscess. The overall incidence of morbidity and mortality were similar to other RAMPS studies. Mitchem et al. [12] evaluated the long-term results of RAMPS among 47 patients who underwent anteroposterior RAMPS. They found that the overall in-hospital mortality rate was 0 %, and that the morbidity rate was 39 % (18 patients). In addition, Chang et al. [13] evaluated the surgical outcome of RAMPS in 24 consecutive patients. They demonstrated that the overall in-hospital mortality rate was 0 % and that the morbidity rate was 37.5 % (9 patients). Previous studies have shown the incidence of the morbidity after conventional distal pancreatomy to be 23–58 % [14, 15]. These findings suggest the safety and feasibility of the RAMPS procedure and indicated that it is not associated with increased morbidity or mortality.

After the median follow-up period was 41.1 months, 1-year and 3-year overall survival rates were 84.1 and 38.6 %, respectively. Median overall survival was 22.6 months. Similar results were observed in the previous RAMPS studies. Table 2 showed the comparison of other published series of RAMPS and standard pancreatosplenectomy. Mitchem et al. reported a median survival period of 26 months and a 5-year overall survival rate of 35 % in 47 patients who underwent anteroposterior RAMPS. In the same study, they reported that 23 patients who underwent RAMPS more than 5 years before the date of last follow-up, had a 5-year survival rate of 30.4 %. RAMPS appeared to offer superior survival in comparison to standard DP at high volume centers; the previously reported 5-year survival rates have ranged from 10–19 %. The first possible reason is that the negative margin rate was higher in the RAMPS procedures than in standard DP. When comparing with previous reports, the negative margin rate was 80–90 % in the previous RAMPS studies, while the negative margin was 70–80 % in the standard DP studies (Table 2). A second possibility is that different numbers of lymph nodes were harvested in the RAMPS and standard DP procedures. Trottman et al. [16] examined 26 cases in which RAMPS and standard resection was performed to identify differences in the clinicopathological outcomes of the patients. They demonstrated a significant difference in the mean number of lymph nodes that were removed in standard resection (4.3) and RAMPS (11.2) (*P* = 0.03). Moreover, La Torre et al. [17] compared the clinicopathological outcomes of 25 patients who underwent RAMPS or standard resection. They too demonstrated a significant difference in the mean number of lymph nodes that were removed in standard resection (16.2) and RAMPS (20.7) (*P* = 0.04).

**Table 2:** Comparison of published data of radical antegrade modular pancreatosplenectomy in other studies

Author (year)	Ref No.	Approach	Number of cases	Morbidity	Mortality	Harvest lymph nodes	Negative surgical margin	Median survival	Five-year survival
								(months)	
Brennan (1996) [4]	4	Standard	34	23 %	0 %	13	68 %	12	14 %
Mitchem (2012) [12]	15	RAMPS	47	39 %	0 %	18.0	91.0 %	26	36 %
Chang (2012) [13]	16	RAMPS	24	37.5 %	0 %	20.9	91.7 %	18.2	NA
Latorre (2013) [17]	20	RAMPS	8	25 %	0 %	20.7	87.5 %	14	26 %
Trottman (2014) [16]	19	RAMPS	6	50 %	0 %	11.2	100 %	NA	NA
Kitagawa (2014) [5]	5	RAMPS	24	58 %	0 %	24	88 %	NA	53 %
Park (2014) [6]	6	Standard	54	22.2 %	0 %	9	85.1 %	NA	12.0 %
		RAMPS	38	18.4 %	0 %	14	89.5 %	NA	40.1 %
Our study (2015)	–	RAMPS	49	41 %	0 %	15	83.7 %	22.6	27 %

The RAMPS procedure should be tested against standard DP in a randomized trial. According to Mitchem et al., a prospective randomized, controlled study should be performed to better determine the long-term outcomes. They calculated that in order to compare the two treatments with 5-year survival rates of 20 and 35 %, a total of 556 patients (228 patients per group) would be required to detect a difference at the 95 % CI. Such numbers would be difficult to obtain in a single institution because resections of adenocarcinoma of the pancreatic body and tail are uncommon in comparison to resections of the pancreatic head.

Special attention is required when interpreting the current results, because several potential limitations are associated with the present study. First, the present study was a retrospective analysis which was performed in a single institution. We cannot deny the possibility that our findings were observed by chance. Second, there was a selection bias in the patients in this series. Surgeons often avoid performing pancreatomy in the some patients, because pancreatectomy itself has a 1–1.5 % mortality rate and a 40–60 % morbidity rate. Thus, the fact that some of the patients in the present study received pancreatectomy could, in and of itself, be a potential bias. In addition, our hospital is a specialized cancer center. The third issue is the follow-up period. In our series, the median follow-up period was approximately 36 months. Our follow-up period may not have been sufficient to allow definite conclusions to be drawn. Considering these limitations, the current results should be validated in other series with a larger number of patients.

## Conclusions

The results indicate that the RAMPS procedure might be safe feasible for the treatment of pancreatic cancer and that it might offer a survival benefit in comparison to standard DP.
